# Differential immunity induced by Omicron sublineages in naïve and vaccine breakthrough infections

**DOI:** 10.1038/s41598-025-07702-2

**Published:** 2025-07-03

**Authors:** Noah Brazer, Mary Kate Morris, Venice Servellita, Miriam Oseguera, Nanami Sumimoto, Prachi Saldhi, Abiodun Foresythe, Jenny Nguyen, Debra A. Wadford, Carl Hanson, Charles Y. Chiu

**Affiliations:** 1 Department of Laboratory Medicine, University of California, San Francisco, San Francisco, CA USA; 2 Viral and Rickettsial Disease Laboratory, California Department of Public Health, Richmond, CA USA; 3 Innovative Genomics Institute, University of California Berkeley, Berkeley, CA 94720 USA; 4 Department of Medicine, University of California San Francisco, San Francisco, CA 94143 USA; 5 Chan-Zuckerberg Biohub, San Francisco, CA 94158 USA

**Keywords:** Omicron, Delta, BA.1, BA.2, BA.4, BA.5, BF, BQ.1, XBB, SARS-CoV-2 variants, COVID-19, Breakthrough infection, Neutralizing antibodies, Vaccine boosting, Genomic analysis, Sequencing, Microbiology techniques, Viral infection, Adaptive immunity, Immune evasion, Vaccines

## Abstract

The emergence of the Omicron variant in late 2021 gave rise to multiple descendent lineages, or sublineages, with progressively increased capacity for antibody evasion. Here we used live virus neutralization assays to quantify and compare homologous (“self”) and cross-neutralizing antibody titers in 170 COVID-19 patients infected with either the Delta variant or an Omicron sublineage (BA.1, BA.2, BA.4/BA.5, BQ.1.1, and XBB.1.5) and 25 uninfected controls who had received the BA.5 bivalent booster vaccine. In control subjects, neutralizing antibody titers against BA.5 and earlier sublineages were significantly higher than against the later BQ.1.1 or XBB.1.5 sublineages, and differences in antibody titers between immunocompetent and immunocompromised individuals were not significant. In patients infected with an Omicron sublineage, induced cross-neutralizing antibody responses were weaker and less durable against later compared to earlier sublineages. Self-neutralizing antibody titers against BQ.1.1 or XBB.1.5 in patients infected with these sublineages were also lower than cross-neutralizing titers against earlier sublineages. Our results suggest that immunological imprinting resulting from prior exposure to SARS-CoV-2 (“original antigenic sin”), whether via natural infection or vaccination, may have impaired neutralizing antibody responses to the later Omicron sublineages. The poorer elicited immunogenicity and increased capacity for antibody evasion of these sublineages explain in part their persistence and ongoing global circulation.

## Introduction

The Severe Acute Respiratory Syndrome Coronavirus 2 (SARS-CoV-2) Omicron (B.1.1.529) variant emerged globally in late 2021^1,2^, and its descendent lineages (“sublineages”) continue to be the predominant strains in circulation as of early 2025^3,4^. Although originating from a single source, serologic evidence to date has suggested that infections from the various Omicron sublineages evoke differential antibody responses, resulting in different levels of homologous (“self”) and cross-neutralization. For example, we previously reported that neutralizing immunity was stronger against BA.2 than BA.1, regardless of the infecting variant^5^. The evolving heterogeneity of Omicron sublineages is also highlighted by BQ.1.1 and XBB, which escaped neutralization from an antibody that was able to broadly neutralize all earlier Omicron sublineages, including BA.1 and BA.4/BA.5, and variants of concern (Alpha, Beta, Gamma, Delta)^6^. In addition, cross-variant neutralization has been reported in vaccine breakthrough infections^5^; however, vaccine breakthrough infections from earlier BA.1, BA.2, and BA.5 sublineages were found to elicit only weak cross-neutralization responses against BQ.1.1 and XBB^7,8^.

The self- and cross-neutralization responses conferred by breakthrough infections from the various Omicron sublineages remains largely unexplored. Here we used live virus neutralization assays to evaluate differential neutralizing antibody responses against Delta and multiple Omicron variants (BA.1, BA.2, BA.5, BF.7, BQ.1.1, and XBB.1.5) in patients with COVID-19.

## Results

### Study cohort

This study included 278 plasma samples collected from July 2021 through June 2023 from 170 COVID-19 patients, of whom 157 (92.4%) were hospitalized, 130 (76.5%) were fully vaccinated with 2 doses of the ancestral SARS-CoV-2 vaccine, 82 (48.2%) boosted with ≥ 1 additional dose of the ancestral vaccine, and 12 (7.1%) boosted with the BA.5 bivalent booster vaccine (Table 1 and Dataset 1). Additionally, the study included 25 plasma samples taken from 25 uninfected control subjects (“controls”), defined as patients who tested negative for SARS-CoV-2 admitted to the hospital for reasons other than COVID-19 who had remnant whole blood available and received the bivalent BA.5 booster vaccine prior to sample collection based on retrospective chart review (Table 2 and Dataset 1). Among the 170 COVID-19 patients, 132 were infected with an Omicron sublineage (77.6%), including 44 BA.1 (25.9%), 13 BA.2 (7.9%), 27 BA.4/BA.5 (15.9%), 5 BF (2.9%), 16 BQ.1 (9.4%), and 27 XBB.1 (15.9%) variants, while the remaining 38 patients were infected with the Delta variant (22.4%). Greater proportions of Delta-infected patients were unvaccinated and presented with severe disease than Omicron-infected patients (Table 3). Ten of the patients were pediatric patients (5.9%), 96 were male (56.5%), and 90 were immunocompetent (52.9%). 87 patients were asymptomatic or presented with mild SARS-CoV-2 respiratory infection (51.2%), most of whom were admitted for reasons other than COVID-19, while 83 (48.8%) had moderately severe or severe infection. Regarding the 25 uninfected controls, all were adults, 15 were female (60.0%), and 18 were immunocompetent (72.0%).

**Table 1:** Clinical and demographic data for the 170 COVID-19 patients in the study.

Characteristic	n	%
Infecting variant		
Delta	38	22.4
BA.1	44	25.9
BA.2	13	7.6
BA.4/BA.5	27	15.9
BF	5	2.9
BQ.1	16	9.4
XBB.1	27	15.9
Age		
< 22	10	5.9
22–64	83	48.8
65 +	77	45.3
Sex		
Female	74	43.5
Male	96	56.5
Immune status		
Immunocompetent	90	52.9
Immunocompromised	80	47.1
Care setting		
Hospitalized	157	92.4
Outpatient	13	7.6
Vaccination status		
Unvaccinated	38	22.4
Partially vaccinated	2	1.2
Unboosted	48	28.2
Single boosted	50	29.4
Double boosted	17	10.0
Triple boosted	3	1.8
BA.5-Bivalent boosted	12	7.1
COVID-19 severity		
Asymptomatic/mild	87	51.2
Moderate/severe	83	48.8

**Table 2:** Clinical and demographic data for the 25 uninfected controls in the study, hospitalized but COVID-19 negative.

Characteristic	n	%
Age		
22–64	11	44.0
65 +	14	56.0
Sex		
Female	15	60.0
Male	10	40.0
Immune status		
Immunocompetent	18	72.0
Immunocompromised	7	28.0

**Table 3:** Clinical and demographic variables stratified by infecting variant for the 170 COVID-19 patients in the study.

	Delta	BA.1	BA.2	BA.4/BA.5	BF	BQ.1	XBB.1
Age							
< 22	0	2	2	3	0	0	3
22–64	21	27	5	9	2	11	8
65 +	17	15	6	15	3	5	16
Sex							
Female	10	22	5	17	1	11	8
Male	28	22	8	10	4	5	19
Immune status							
Immunocompetent	22	27	6	11	3	6	15
Immunocompromised	16	17	7	16	2	10	12
Care setting							
Hospitalized	37	33	12	27	5	16	27
Outpatient	1	11	1	0	0	0	0
Vaccine status							
Unvaccinated	13	11	2	0	2	0	6
Vaccinated (without BA.5 booster)	25	33	10	23	3	15	11
BA.5-bivalent boosted	0	0	1	4	0	1	10
Severity of infection							
Asymptomatic/mild	9	23	9	10	3	9	20
Moderate/severe	29	21	4	17	2	7	7

**Dataset 1** Deidentified patient and control data. A total of 278 plasma samples from 170 COVID-19 patients (first tab) and 25 plasma samples from 25 uninfected vaccinated control subjects (second tab) were included in this study.

### Neutralizing antibody responses in boosted uninfected controls

**Fig. 1:**
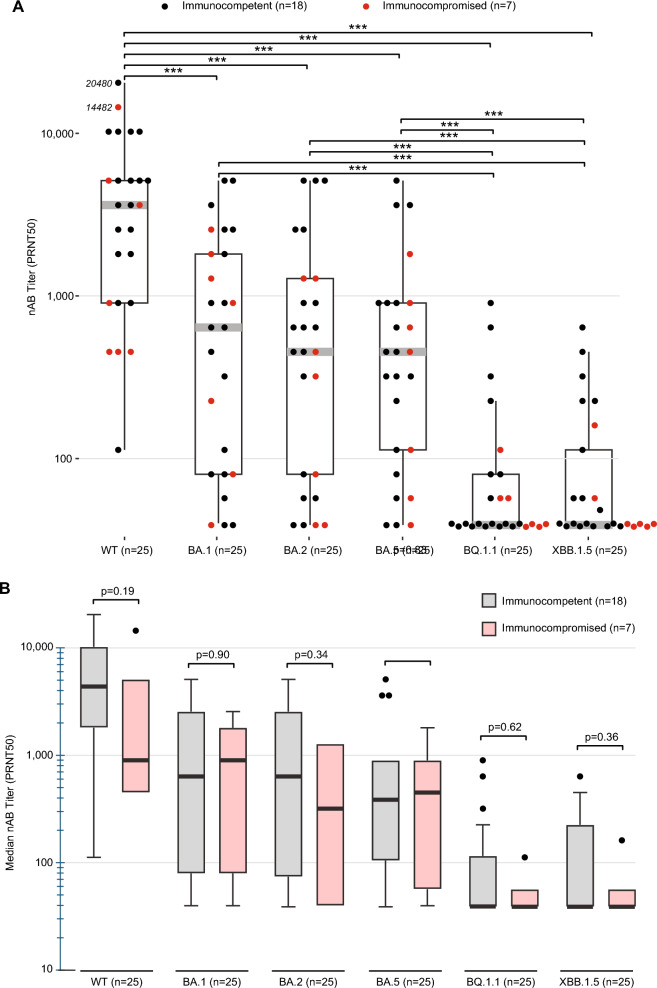
Neutralizing antibody responses in uninfected adult controls. (**A**) Box and whisker plots showing neutralizing antibody titers against the SARS-CoV-2 ancestral strain (“WT”) and Omicron sublineages in a control cohort of hospitalized patients without COVID-19 who had previously received the BA.5 bivalent booster vaccine (n = 25). Black points denote neutralizing antibody responses in immunocompetent control patients and red points in immunocompromised control patients. The gray bar denotes the median, the boundaries of the box the upper and lower quartiles of the median, and the whiskers the minimum and maximum values. ***, *p* < 0.001. (**B**) Median neutralizing antibody titers against each sublineage in immunocompetent versus immunocompromised control patients. The upper error bars denote the standard error of the median.

In the control cohort of uninfected subjects who had received the bivalent BA.5 booster vaccine, neutralizing antibody titers against the wild-type ancestral strain (“WT”) were significantly higher than those against all the Omicron sublineages (*p* < 0.001) (Fig. 1A). Neutralizing antibody titers against BA.1, BA.2, and BA.5 were significantly higher than those against BQ.1.1 or XBB.1.5 (*p* < 0.001). Overall differences in neutralizing antibody titers against all variants between immunocompetent and immunocompromised controls were non-significant (Fig. 1B).

### Self-neutralizing antibody responses in COVID-19 patients

**Fig. 2:**
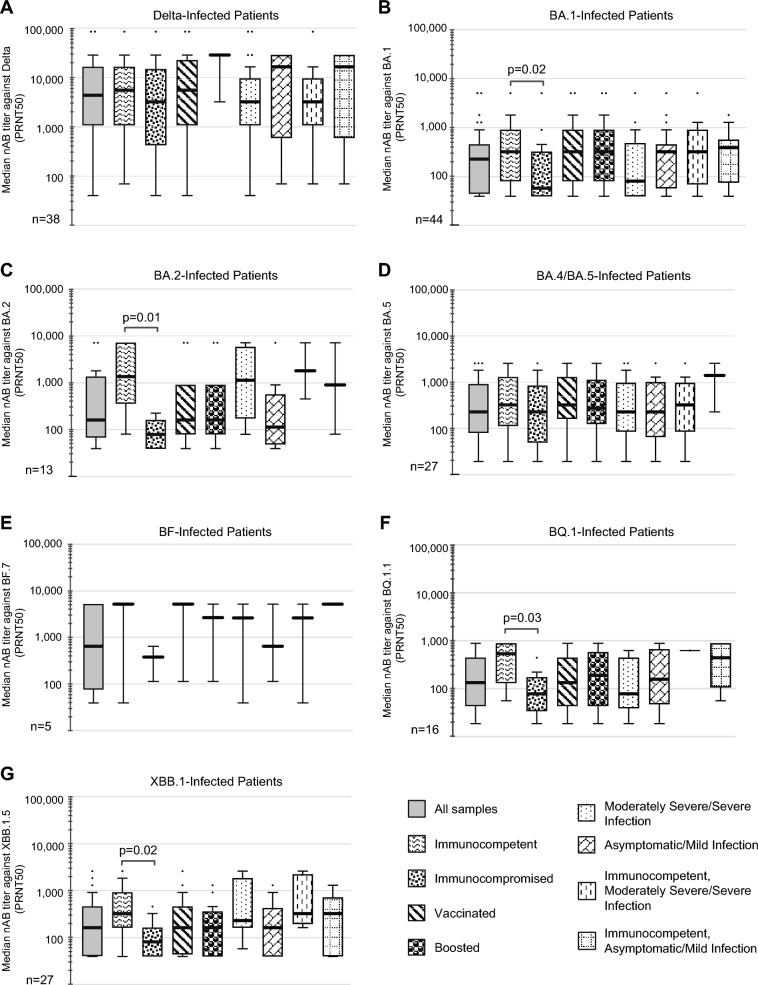
Homologous (“self”) neutralizing antibody responses by COVID-19 patient subgroup. Box and whisker plots showing median self-neutralizing antibody titers against (**A**) Delta, (**B**) BA.1, (**C**) BA.2, (**D**) BA.5, (**E**) BF.7, (**F**) BQ.1.1, and (**G**) XBB.1.5 in patients infected with Delta, BA.1, BA.2, BA.4 or BA.5, BF, BQ.1, and XBB.1 sublineages, respectively, are shown. The thicker black bar denotes the median, the boundaries of the box represent the upper and lower quartiles of the median, and the whiskers indicate ± 1.5 times the interquartile range according to the Tukey method.

**Fig. 3:**
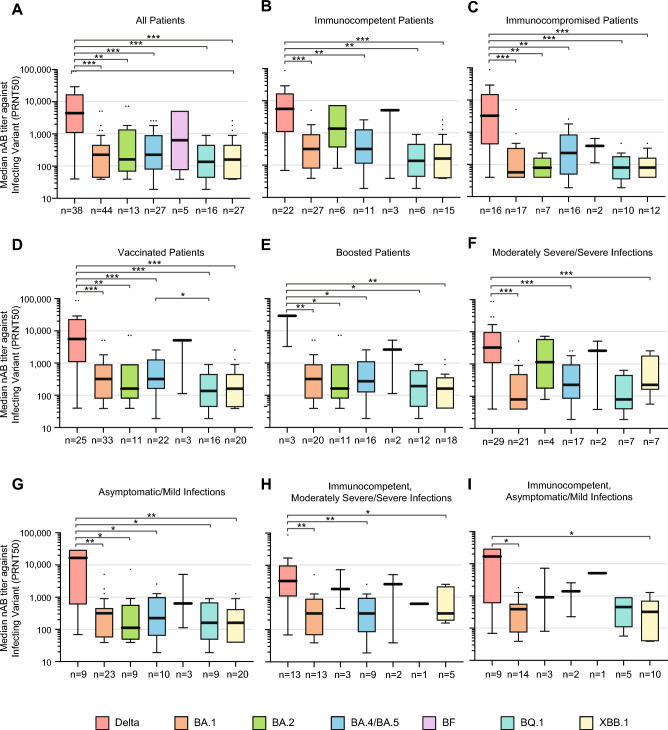
Homologous (“self”) neutralizing antibody responses by infecting variant or sublineage. Box and whisker plots showing median self-neutralizing antibody titers against the respective infecting variant or sublineage in (**A**) all patients, (**B**) immunocompetent patients, (**C**) immunocompromised patients, (**D**) fully vaccinated patients, including those who had received a booster vaccine targeting the SARS-CoV-2 ancestral strain, (**E**) boosted patients only, (**F**) 170 with moderately severe or severe infection, (**G**) patients with asymptomatic or mild infection, (**H**) immunocompetent patients with moderately severe or severe infection, (**I**) immunocompetent patients with asymptomatic or mild infection, are shown. The thicker black bar denotes the median, the boundaries of the box represent the upper and lower quartiles of the median, and the whiskers indicate  ± 1.5 times the interquartile range according to the Tukey method. *, *p* < 0.05; **, *p* < 0.01; ***, *p* < 0.001.

We compared self-neutralizing antibody responses against the infecting variant or sublineage in patients stratified by immune status, vaccination status, and disease severity. Immunocompetent patients infected with BA.1, BA.2, BQ.1, or XBB.1 exhibited significantly higher self-neutralizing antibody titers than immunocompromised patients infected with the respective variant (*p* = 0.01–0.03) (Fig. 2), although this association was not significant for patients infected with Delta, BA.4/BA.5, or BF. Self-neutralizing antibody titers from Delta infections were significantly higher than those from Omicron variant infections, excluding BF, for all patients overall (*p* < 0.01) as well as for nearly all patient subgroups stratified by immunocompromised status, vaccination status, or COVID-19 severity (*p* < 0.001–0.024) (Fig. 3).

### Cross-neutralizing antibody responses in COVID-19 patients

**Fig. 4:**
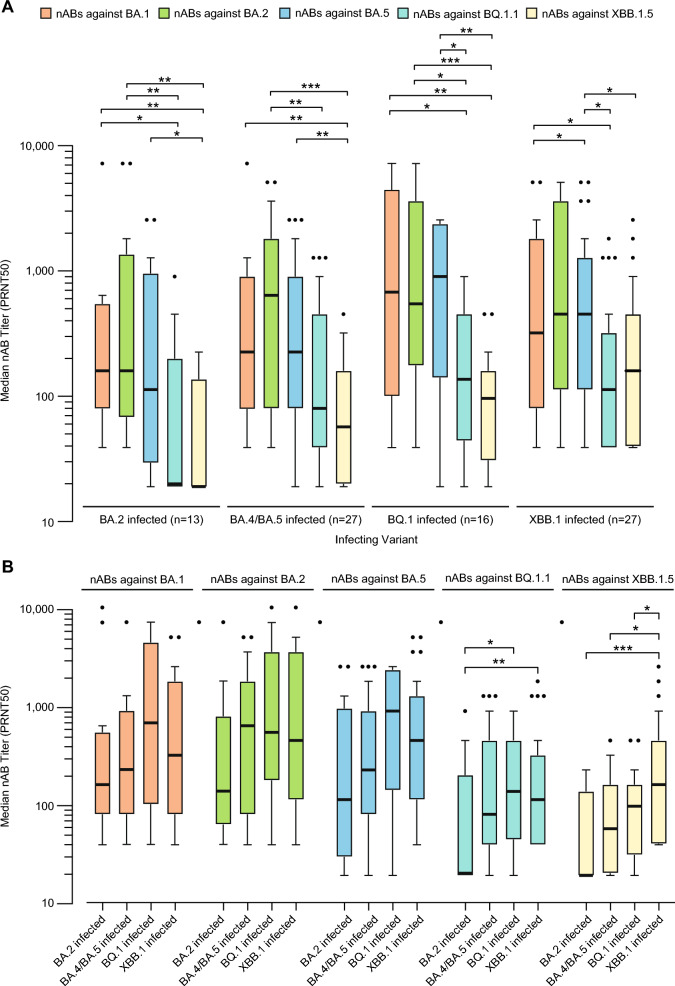
Homologous (“self”) and cross-neutralizing antibody responses. (**A**) Box and whisker plots showing median neutralizing antibody titers against BA.2, BA.5, BQ.1.1, and XBB.1.5 grouped by infecting sublineage. (**B**) Box and whisker plots showing median neutralizing antibody titers for BA.2-infected, BA.4/BA.5-infected, BQ.1-infected, and XBB.1-infected patients grouped by sublineage of the cultured isolate used in the live virus neutralization assay. The thicker black bar denotes the median, the boundaries of the box represent the upper and lower quartiles of the median, and the whiskers indicate ± 1.5 times the interquartile range according to the Tukey method. *, *p* < 0.05; **, *p* < 0.01; ***, *p* < 0.001.

For each infecting sublineage, we compared neutralization antibody titers against cultured isolates representing the BA.1, BA.2, BA.5, BQ.1.1, and XBB.1.5 Omicron sublineages (Fig. 4A). Overall, cross-neutralizing titers were robust against earlier Omicron sublineages, with PRNT50 values > 1:100, but were much weaker against later sublineages, especially if these sublineages emerged after the infecting sublineage. For BA.2 infections, cross-neutralizing antibody titers were significantly lower against BQ.1.1 than BA.1 (*p* = 0.011) and BA.2 (*p* = 0.008), while cross-neutralizing titers were significantly lower against XBB.1.5 than BA.1 (*p* = 0.005), BA.2 (*p* = 0.004), and BA.5 (*p* = 0.042). For BA.4/BA.5 infections, cross-neutralizing titers were significantly lower against BQ.1.1 than BA.2 (*p* = 0.009), while cross-neutralizing titers were significantly lower against XBB.1.1 than BA.1 (*p* = 0.003), BA.2 (*p* < 0.001), and BA.5 (*p* = 0.004). For BQ.1 infections, self-neutralization titers were significantly lower against BQ.1.1 than cross-neutralizing titers against BA.1 (*p* = 0.039), BA.2 (*p* = 0.023), and BA.5 (*p* = 0.017), while cross-neutralizing titers were significantly lower against XBB.1.5 than BA.1 (*p* = 0.003), BA.2 (*p* < 0.001), and BA.5 (*p* = 0.003). In XBB.1 infections, cross-neutralizing antibody titers were significantly lower against BQ.1.1 than BA.2 (*p* = 0.012) and BA.4/BA.5 (*p* = 0.013), while self-neutralizing antibody titers against XBB.1.5 were significantly lower than cross-neutralizing antibody titers against BA.2 (*p* = 0.033) and BA.4/BA.5 (*p* = 0.040),

We compared relative neutralizing antibody titers in patients infected with different Omicron sublineages. Neutralizing antibody titers against BQ.1.1 were significantly higher in BQ.1 and XBB.1 infections compared to those in BA.2 infections (*p* = 0.046 and *p* = 0.009, respectively) (Fig. 4B). Neutralizing antibody titers against XBB.1.5 were significantly higher in XBB.1 infections compared to those in BA.2 infections (*p* < 0.001), BA.4/BA.5 infections (*p* = 0.016), and BQ.1 infections (*p* = 0.046).

### Effect of prior infection and vaccination on neutralizing antibody responses

**Fig. 5:**
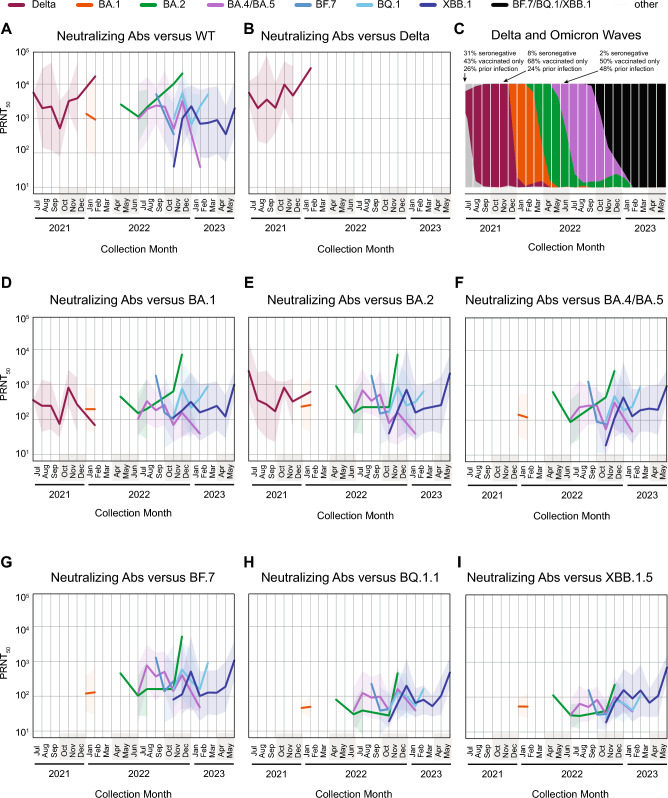
Neutralizing antibody titers against wild-type, Delta, and Omicron lineages in COVID-19 patients. For each infecting lineage (colored lines), mean titers against wild-type (**A**), Delta (**B**), and the Omicron sublineages (**D**–**I**) are plotted by collection month. The shaded regions represent the standard deviation. (**C**) Plot of variant proportions obtained from genomic surveillance for SARS-CoV-2 in the United States by the Centers for Disease Control and Prevention (CDC)^2,33^. The plot is annotated with COVID-19 seroprevalence data collected by the California Department of Public Health^9^. Note that there is a gap in sample collection from February to April 2022, as public health surveillance efforts to control the spread of cases locally were prioritized at that time.

Next, we plotted mean neutralizing antibody titers against wild-type, Delta, and Omicron lineages in infected patients by collection month (Fig. 5A,B,D–I).@@ We also plotted in parallel the emergence of the Delta and Omicron sublineage waves by collection month and annotated the graph with population-level statewide seroprevalence data (Fig. 5C)^9^. At the onset of the Omicron BA.1 and BA.4/BA.5 waves, only 8% and 2% of the population, respectively, were seronegative. This observation is consistent with the finding of robust neutralizing antibody titers (PRNT50 ≥ 10^3^) against WT across all variant and sublineage infections (Fig. 5A). Notably, whereas Delta-infected patients generated robust neutralizing Abs versus Delta (Fig. 5B), this was not generally the case for patients infected with Omicron sublineages. Among these sublineages, robust neutralizing antibody titers were only observed for BA-2 infected patients against BA.1, BA.2, BA.4/BA.5, and BF.7 (Fig. 5D–G) and for XBB.1 infected patients against the other lineages (Fig. 5D–I).

### Longitudinal analyses of neutralizing antibody responses

**Fig. 6:**
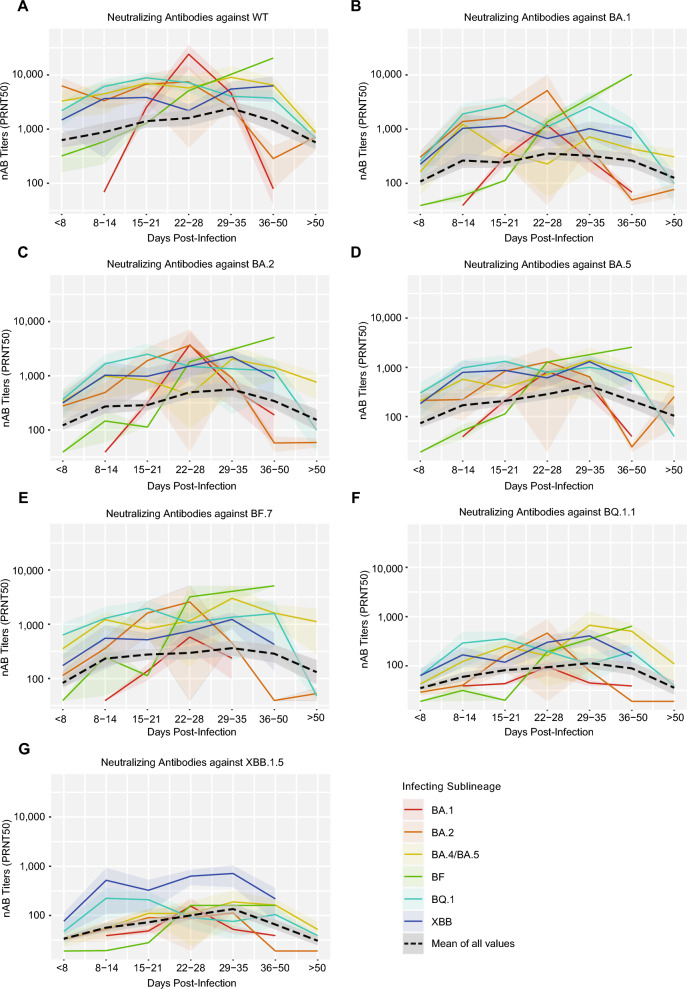
Longitudinal neutralizing antibody responses. Plot of median homologous (“self”) or cross-neutralizing neutralizing antibody titers against (**A**) the wild-type ancestral strain (“WT”), (**B**) BA.1, (**C**) BA.2, (**D**) BA.5, (**E**) BF.7, (**F**) BQ.1.1 and (**G**) XBB.1.5 binned into serial time intervals (in days) post-infection as determined based on date of symptom onset or first positive SARS-CoV-2 PCR test. The shaded regions represent the standard deviation at each time interval. The dotted line shows the mean of all titers at the respective time interval.

Samples collected at various days post-infection based on the date of symptom onset or first positive SARS-CoV-2 PCR test were available for longitudinal analyses of neutralizing antibody titers from 132 patients infected with Omicron sublineages from January 2022 through June 2023 (Fig. 6). In BA.1 and BF infections, neutralizing antibody titers increased more slowly and to lower peak levels over time compared to infections from other Omicron variants. In addition, neutralizing antibody titers from infection by earlier Omicron variants (BA.1 and BA.2) waned more quickly than those from infection by later Omicron variants (BA.4/BA.5, BF, BQ.1, and XBB.1).

## Discussion

This study employed live virus assays to quantify and compare neutralizing antibody titers in a cohort of 170 patients infected with Delta, BA.1, BA.2, BA.4/BA.5, BF, BQ.1, or XBB.1, most (94%) of whom were hospitalized, and in 25 uninfected controls who had previously received the BA.5 bivalent booster vaccine. Uninfected controls who previously received the bivalent BA.5 booster vaccine had significantly higher neutralizing antibody titers against BA.1, BA.2, and BA.5 compared to those against the later BQ.1.1 or XBB.1.5 sublineages (Fig. 1A). We also found that self-neutralizing antibody titers from Delta infections were significantly higher than those from Omicron sublineage infections, both overall and in the immunocompetent and moderately severe to severe infection patient subgroups (Fig. 3). Similarly, infection with BA.4/BA.5 or earlier sublineages, such as BA.1 and BA.2, elicited weak and less durable cross-neutralizing antibody responses against later sublineages, including BQ.1.1 and XBB.1.5 (Fig. 4A). Of note, while self-neutralizing antibody titers against BQ.1 and XBB.1 tended to be higher than cross-neutralizing antibody titers associated with infections from other sublineages, these titers remained relatively low. Both BQ.1 and XBB.1 infections resulted in higher cross-neutralizing antibody titers against BA.1, BA.2, or BA.5 than self-neutralizing antibody titers against either BQ.1.1 or XBB.1.5. Taken together, these findings suggest that the later SARS-CoV-2 Omicron variant sublineages have progressively evolved to exhibit poorer immunogenicity and increased capacity for antibody evasion.

Our finding of consistently lower self-neutralizing antibody responses from Omicron variant sublineage infections compared to those from Delta infections points to an important property of Omicron variants that may explain their persistence as the dominating SARS-CoV-2 strain. This observed phenomenon could be partially due to “original antigenic sin” resulting from prior exposures to SARS-CoV-2 variants with antigenic similarity to Delta, whether through natural infection or vaccination. These prior exposures elicit strong responses to Delta and earlier variants^10,11^, but may impair responses to future variants such as Omicron and its sublineages due to immunological imprinting^12–14^. Imprinting as a mechanism by which later Omicron sublineages evade antibody neutralization is also supported by our findings that prior infection from and/or vaccination against earlier variants, even hybrid immunity, failed to generate robust neutralizing antibody titers against later variants and thus were likely not protective against subsequent infection, despite a population seroprevalence of 92% at the onset of the Omicron sublineage waves (Fig. 5C). Another non-mutually exclusive explanation for the increased antibody evasion of the later Omicron sublineages may be structural changes in the spike protein resulting from ongoing mutations that render them partially or completely resistant to neutralization^15,16^.

Prior to the development of the BA.5 bivalent booster vaccine, all COVID-19 vaccines were developed against the ancestral strain, which is antigenically more like Delta than the Omicron sublineages^5^. It may be worth exploring whether exclusion of the non-circulating strains such as the ancestral strain from the vaccine would mitigate the effect of immunological imprinting and thus restore vaccine-induced immunogenicity. Higher self-neutralizing antibody titers with Delta compared to Omicron infections may also be related in part to higher infectious viral loads and longer periods of viral shedding with Delta^17^, thereby eliciting stronger and more durable antibody responses.

No significant differences in neutralizing antibody titers were observed between uninfected immunocompetent and immunocompromised controls who had previously received the BA.5 bivalent booster vaccine. Improved or de novo immune responses after administration of a booster vaccine have been reported for most immunocompromised individuals despite their immunosuppressed status^18^. In addition, among immunocompromised individuals, those who were vaccinated exhibited higher immune responses following infection with BA.1 or BA.2 than those who were unvaccinated^5,19^. Follow-up studies are warranted to determine whether our finding of comparable neutralizing immune responses against Omicron sublineage infections between immunocompetent and immunocompromised patients holds true with larger sample sizes.

There are some limitations to this study. Only remnant SARS-CoV-2-positive biobanked samples were included, and hence only a limited number of convalescent samples were available for analysis. The stratification by infecting variant, severity, and immunocompromised status resulted in subgroups that were underpowered to detect small effects. Vaccination status and other clinical metadata were gathered from electronic medical records retrospectively rather than prospectively. Thus, any error in the electronic medical records would generate inaccuracies in the extracted clinical metadata. Additionally, documentation of prior SARS-CoV-2 infections is limited, especially in cases of asymptomatic infection, which may affect the neutralization titers in both infected patients and uninfected controls. Finally, the results from the study may not be generalizable to individuals with mild or asymptomatic infections or the pediatric population, as study subjects consisted of predominantly hospitalized patients (92.4%) and only 4.7% were children.

All sublineage infections, including from BQ.1 and XBB.1, resulted in poor neutralizing antibody responses against BQ.1.1 and XBB.1.5. Although previous studies in outpatients have shown that BA.4/BA.5 and earlier sublineage infections elicit poor neutralizing antibody responses against BQ.1.1 and XBB1.5^15,20–22^, here we reproduce these results in a cohort of primarily hospitalized patients, both immunocompromised and immunocompetent, and exhibiting various degrees of COVID-19 severity. Here we also confirm that BQ.1 and XBB.1 infections elicit poor self-neutralizing antibody responses^15,21^. Conformational changes in the spike proteins of BQ.1 and XBB.1 may afford these variants greater capacity for immune escape from vaccination or natural infection^22,23^. Additionally, heterogeneity among BQ.1 and XBB.1 sublineages may explain in part the increased antibody evasion^24^. Ten (7.1%) of the 12 COVID-19 patients in our study who had received the bivalent BA.5 booster vaccine were infected with XBB.1 and exhibited poor neutralizing antibody responses despite vaccination. Considering the rapidly evolving nature of SARS-CoV-2, it is crucial to understand the dynamic drivers of neutralizing immunity to inform vaccine development and anticipation of the emergence and pathogenicity of future variants.

## Methods

### Human subjects

The human subjects in this study included 170 with COVID-19 and 25 controls without COVID-19. The 170 subjects with COVID-19 included patients at University of California, San Francisco (UCSF) and individuals enrolled in the UMPIRE (UCSF EMPloyee and community member Immune Response) study, a prospective longitudinal study of immune responses to COVID-19 vaccination^11^. The controls consisted of UCSF patients hospitalized for reasons other than COVID-19 and who had previously received the BA.5 bivalent booster. For UCSF patients, remnant plasma samples were biobanked and collection of metadata performed with waiver of informed consent and using protocols approved by the UCSF Institutional Review Board (IRB) (protocol numbers 10-01116 and 11-05519). For UMPIRE study subjects, informed consent for participation and collection of metadata and plasma samples was obtained using a protocol approved by the UCSF IRB (protocol number 20-33083). All research was performed in accordance with relevant guidelines and regulations as established by the UCSF IRB and Declaration of Helsinki.

### Cell lines

For SARS-CoV-2 isolation in cell cultures and performing the live virus assays, Vero E6-TMPRSS2-T2A-ACE2 and Vero-81 cell lines derived from African green monkey kidney cells were cultured at 37 °C in Modified Eagle Medium (MEM) supplemented with 1× penicillin–streptomycin (Gibco), glutamine (Gibco), and 10% fetal calf serum (Hyclone). The Vero E6-TMPRSS2-T2A-ACE2 cell line was also supplemented with 10ug/mL puromycin. The Vero CCL-81 and Vero E6-TMPRSS2-T2A-ACE2 cell lines was obtained from ATCC and BEI Resources, respectively. Both cell lines were authenticated by the manufacturer. The authentication of the Vero E6-TMPRSS2-T2A-ACE2 cell line included confirmation of ACE2 and TMPRSS2 expression by indirect fluorescent antibody assay, confirmation of African green monkey origin by multiplex PCR amplification of the cytochrome C oxidase I gene, and exclusion of Mycoplasma contamination by PCR.

### Human sample and data collection

Blood samples for study subjects were collected as follows. For COVID-19 patients at UCSF, including both children and adults, remnant plasma samples were collected daily from the UCSF Clinical Laboratories based on availability. Retrospective medical chart reviews were done to obtain relevant demographic and clinical metadata. For subjects in the UMPIRE study, plasma samples were collected at approximately 1, 2, and 6-month intervals following a vaccine breakthrough infection. Metadata from UMPIRE participants were obtained through secure online research surveys (Qualtrics, Inc.) performed at enrollment and at each blood draw. For controls, remnant plasma samples were collected and metadata extracted through retrospective chart review. All collected plasma samples were heat inactivated at 56 °C for 30 min prior to use in the live virus assays.

### Clinical chart review

The criteria for a moderately severe infection included hospitalization for COVID-19 pneumonia with an oxygen requirement of > 2 L of oxygen by nasal cannula or development of a non-respiratory complication of the disease (e.g. acute renal injury, diarrhea with electrolyte disturbances, necrosis of the extremities, encephalopathy). The criteria for a severe infection included COVID-19 pneumonia with severe hypoxemia with an oxygen requirement of > 6 L, including the need for CPAP (continuous positive airway pressure), BiPAP (bilevel positive airway pressure), or intubation with mechanical ventilation, COVID-19 associated end-organ failure, and/or death. Outpatients and hospitalized patients not meeting criteria for moderately severe or severe infection were classified as having a mild infection or asymptomatic infection if they did not exhibit any COVID-19 related symptoms.

The criteria for classification as immunocompromised included patients on immunosuppressive therapy due to active malignancies or solid organ or bone marrow transplantation and patients with any congenital, infectious, or other type of disease associated with a severe immunodeficiency.

### SARS-CoV-2 whole-genome sequencing

Viral whole-genome sequencing of SARS-CoV-2 for 136 of 170 patients for variant or sublineage identification was performed as previously described^11^ (Dataset 1). For the remaining 34 patients, variant or sublineage identification was inferred based on the date of symptom onset. Remnant nasopharyngeal (NP) and/or oropharyngeal swab samples collected in universal transport media or viral transport media were diluted with DNA/RNA shield (Zymo Research, # R1100-250) in a 1:1 ratio (100 μl primary sample + 100 μl shield) prior to viral RNA extraction. The Omega BioTek MagBind Viral DNA/RNA Kit (Omega Biotek, # M6246-03) and the KingFisherTM Flex Purification System with a 96 deep-well head (Thermo Fisher, 5400630) were used for viral RNA extraction. Extracted RNA was reverse transcribed to complementary DNA and tiling multiplexed amplicon PCR was performed using Artic version 3 and/or VarSkip SARS-CoV-2 primers according to a previously published protocol (Quick et al. 2017). Adapter ligation was performed using the NEBNext^®^ ARTIC SARS-CoV-2 FS Library Prep Kit (Illumina^®^) (New England Biolabs, # E7658L). Libraries were barcoded using NEBNext Multiplex Oligos for Illumina (96 unique dual-index primer pairs) (New England Biolabs, # E6440L) and purified with AMPure XP (Beckman-Coulter, #63880). Amplicon libraries were then sequenced on either Illumina MiSeq or NextSeq 550 as 2 × 150 base pair paired-end reads.

### Genome assembly, variant identification and mutation analysis

Raw sequencing data were simultaneously demultiplexed and converted to FASTQ files and screened for SARS-CoV-2 sequences using BLASTn (BLAST + package 2.9.0). Reads containing adapters, the ARTIC and/or VarSkip primer sequences, and low-quality reads were filtered using BBDuk (version 38.87) and then mapped to the Wuhan-Hu-1 SARS-CoV-2 reference genome (National Center for Biotechnology Information (NCBI) GenBank accession number NC_045512.2) using BBMap (version 38.87) as previously described^25^. Consensus sequences were generated using iVar (version 1.3.1)^26^ and lineages were assigned using Pangolin (version 3.1.17)^27^ and NextClade (version 1.11.0)^28^ software.

### SARS-CoV-2 isolation in cell cultures

All SARS-CoV-2 lineages were isolated from de-identified patient NP swab samples sent to the California Department of Public Health from hospitals in California for surveillance purposes^29^. To isolate the Delta variant, 200 μl of a nasopharyngeal (NP) sample that was previously identified as Delta was diluted 1:3 in PBS supplemented with 0.75% bovine serum albumin (BSA-PBS) and added to confluent Vero-81 cells in a T25 flask. Following a 1-h absorption period, additional media was added, and the flask was incubated at 37 °C with 5% CO2 with daily monitoring for cytopathic effect (CPE). When 50% CPE was detected, the contents were collected, clarified by centrifugation, and stored at −80 °C as passage 0 stock. A passaged stock (p1) of Delta was made by inoculation of Vero-81 confluent T150 flasks with 1:10 diluted p0 stock and harvested at approximately 50% CPE. Omicron viral stock for sublineages of interest was similarly produced from sequence-confirmed NP samples cultured in Vero E6-TMPRSS2-T2A-ACE2 cells. All viral stocks were sequenced to confirm lineage and the TCID50 was determined by titration.

### Live virus neutralization assay

CPE endpoint neutralization assays were done following the limiting dilution model using p1 stocks of each SARS-CoV-2 lineage in Vero E6-TMPRSS2-T2A-ACE2 cells. Patient plasma was diluted 1:10 in 0.75% bovine serum albumin-phosphate buffered saline (BSA-PBS) and heat inactivated at 56C for 30 min. Serial twofold dilution of plasma were made in BSA-PBS. Plasma dilutions were mixed with 100 TCID50 (tissue culture infective dose 50, or the dose at which 50% of inoculated cells in culture are infected) of each virus diluted in BSA-PBS at a 1:1 ratio and incubated for 1 h at 37 °C. Final plasma dilutions in plasma-virus mixture ranged from 1:40 to 1:20,480. 100 μl of the plasma-virus mixtures was added in duplicate to flat bottom 96-well plates pre-seeded with Vero E6-TMPRSS2-T2A-ACE2 cells at a density of 2.5 × 10^4^/well and incubated in a 37 °C incubator with 5% CO2 until consistent CPE was seen in the virus control (no neutralizing plasma added) wells. Positive and negative controls were included as well as cell control wells and a viral back titration was used to verify TCID50 viral input. Individual wells were scored for CPE as having a binary outcome of “infection” or “no infection” and the ID50 (inhibitory dose 50, the concentration of plasma needed to inhibit virus-induced CPE by 50%), was calculated using the Spearman-Karber method. All steps were done in a Biosafety Level 3 (BSL-3) lab using approved protocols.

### Quantification and statistical analysis

Statistical analyses and data visualization were performed using R (version 4.0.2)^30^. Comparisons between different neutralizing antibody titer groups were conducted using median values. Statistical details of each comparison can be found in the main text of the study as well as in the figures themselves. Significance testing was performed using the Wilcoxon-Mann–Whitney U test for unpaired samples. Plots were generated using the ggplot2 package (version 3.3.5)^31^ in R or Prism 10 software (GraphPad Software). All statistical tests were conducted as two-sided at the 0.05 significance level. Exact values of n are listed in the main text of the paper for each portion of the study, where n represents the number of SARS-CoV-2 infected individuals. Subjects were excluded if they were identified to be infected with a variant that was not Delta, BA.1, BA.2, BA.4, BA.5, BF, BQ.1, or XBB. For variant comparisons, BA.4 and BA.5 were combined to increase sample numbers and because BA.4 and BA.5 emerged at the same time, circulated in the same wave, and have identical spike proteins^32^.

## Data Availability

SARS-CoV-2 viral consensus genome sequences for all samples, clinical and demographic data for COVID-19 patients and controls, and serologic data have been deposited a publicly accessible Zenodo database repository (10.5281/zenodo.15129186).

## References

[1] Elbe, S. & Buckland-Merrett, G. Data, disease and diplomacy: GISAID’s innovative contribution to global health. *Glob. Chall.* **1**, 33–46. 10.1002/gch2.1018 (2017). 31565258 10.1002/gch2.1018 PMC6607375

[2] Lambrou, A. S. et al. Genomic Surveillance for SARS-CoV-2 Variants: Predominance of the Delta (B.1.617.2) and Omicron (B.1.1.529) Variants—United States, June 2021–January 2022. *MMWR Morb. Mortal Wkly. Rep.* **71**, 206–211. 10.15585/mmwr.mm7106a4 (2022). 35143464 10.15585/mmwr.mm7106a4 PMC8830620

[3] Ma, K. C. et al. Genomic surveillance for SARS-CoV-2 variants: Circulation of Omicron XBB and JN.1 lineages—United States, May 2023–September 2024. *MMWR Morb. Mortal Wkly. Rep.* **73**, 938–945 (2024). 39446667 10.15585/mmwr.mm7342a1 PMC11500842

[4] World Health Organization. COVID-19 variants | WHO COVID-19 dashboard, (World Health Organization, 2025); https://data.who.int/dashboards/covid19/variants.

[5] Brazer, N. et al. Neutralizing immunity induced against the Omicron BA.1 and BA.2 variants in vaccine breakthrough infections. *J. Infect. Dis.* **226**, 1688–1698. 10.1093/infdis/jiac384 (2022). 36134603 10.1093/infdis/jiac384 PMC9619439

[6] Sun, H. et al. Structural basis for broad neutralization of human antibody against Omicron sublineages and evasion by XBB variant. *J. Virol.* **97**, e0113723. 10.1128/jvi.01137-23 (2023). 37855619 10.1128/jvi.01137-23 PMC10688377

[7] Yang, J. et al. Low levels of neutralizing antibodies against XBB Omicron subvariants after BA.5 infection. *Signal Transduct. Target Ther.* **8**, 252. 10.1038/s41392-023-01495-4 (2023). 37336889 10.1038/s41392-023-01495-4 PMC10279763

[8] Zhu, K. L. et al. Durability of neutralization against Omicron subvariants after vaccination and breakthrough infection. *Cell Rep.* **42**, 112075. 10.1016/j.celrep.2023.112075 (2023). 36774551 10.1016/j.celrep.2023.112075 PMC9906998

[9] California Department of Public Health. COVID-19 Seroprevalence Data, (California Department of Public Health, 2025); https://www.cdph.ca.gov/Programs/CID/DCDC/pages/COVID-19/sero-prevalence-COVID-19-data.aspx.

[10] Perez-Then, E. et al. Neutralizing antibodies against the SARS-CoV-2 Delta and Omicron variants following heterologous CoronaVac plus BNT162b2 booster vaccination. *Nat. Med.* **28**, 481–485. 10.1038/s41591-022-01705-6 (2022). 35051990 10.1038/s41591-022-01705-6 PMC8938264

[11] Servellita, V. et al. Neutralizing immunity in vaccine breakthrough infections from the SARS-CoV-2 Omicron and Delta variants. *Cell* **185**, 1539–1548. 10.1016/j.cell.2022.03.019 (2022). 35429436 10.1016/j.cell.2022.03.019 PMC8930394

[12] Cao, Y. et al. Imprinted SARS-CoV-2 humoral immunity induces convergent Omicron RBD evolution. *Nature* **614**, 521–529. 10.1038/s41586-022-05644-7 (2023). 36535326 10.1038/s41586-022-05644-7 PMC9931576

[13] Koutsakos, M. & Ellebedy, A. H. Immunological imprinting: Understanding COVID-19. *Immunity* **56**, 909–913. 10.1016/j.immuni.2023.04.012 (2023). 37105169 10.1016/j.immuni.2023.04.012 PMC10113596

[14] Pusnik, J. et al. Vaccination impairs de novo immune response to omicron breakthrough infection, a precondition for the original antigenic sin. *Nat. Commun.* **15**, 3102. 10.1038/s41467-024-47451-w (2024). 38600072 10.1038/s41467-024-47451-w PMC11006949

[15] Wang, Q. et al. Alarming antibody evasion properties of rising SARS-CoV-2 BQ and XBB subvariants. *Cell* **186**, 279–286. 10.1016/j.cell.2022.12.018 (2023). 36580913 10.1016/j.cell.2022.12.018 PMC9747694

[16] Cao, Y. et al. BA.2.12.1, BA.4 and BA.5 escape antibodies elicited by Omicron infection. *Nature* **608**, 593–602. 10.1038/s41586-022-04980-y (2022). 35714668 10.1038/s41586-022-04980-y PMC9385493

[17] Lunt, R. et al. The impact of vaccination and SARS-CoV-2 variants on the virological response to SARS-CoV-2 infections during the Alpha, Delta, and Omicron waves in England. *J. Infect.* **88**, 21–29. 10.1016/j.jinf.2023.10.016 (2024). 37926118 10.1016/j.jinf.2023.10.016

[18] Parker, E. P. K. et al. Response to additional COVID-19 vaccine doses in people who are immunocompromised: a rapid review. *Lancet Glob. Health* **10**, e326–e328. 10.1016/S2214-109X(21)00593-3 (2022). 35180408 10.1016/S2214-109X(21)00593-3 PMC8846615

[19] Ferreira, V. H. et al. Homotypic and heterotypic immune responses to Omicron variant in immunocompromised patients in diverse clinical settings. *Nat. Commun.* **13**, 4489. 10.1038/s41467-022-32235-x (2022). 35927279 10.1038/s41467-022-32235-x PMC9352686

[20] Faraone, J. N. et al. Neutralization escape of Omicron XBB, BR2, and BA2320 subvariants. *Cell Rep. Med.* **4**, 101049. 10.1016/j.xcrm.2023.101049 (2023). 37148877 10.1016/j.xcrm.2023.101049 PMC10213954

[21] Miller, J. et al. Substantial neutralization escape by SARS-CoV-2 Omicron variants BQ11 and XBB1. *N. Engl. J. Med.* **388**, 662–664. 10.1056/NEJMc2214314 (2023). 36652339 10.1056/NEJMc2214314 PMC9878581

[22] Qu, P. et al. Enhanced evasion of neutralizing antibody response by Omicron XBB.1.5, CH.1.1, and CA.3.1 variants. *Cell Rep.* **42**, 112443. 10.1016/j.celrep.2023.112443 (2023). 37104089 10.1016/j.celrep.2023.112443 PMC10279473

[23] Ju, B. et al. Omicron BQ.1.1 and XBB.1 unprecedentedly escape broadly neutralizing antibodies elicited by prototype vaccination. *Cell Rep* **42**, 112532. 10.1016/j.celrep.2023.112532 (2023). 37219999 10.1016/j.celrep.2023.112532 PMC10201307

[24] Osterman, A. et al. Automated antigen assays display a high heterogeneity for the detection of SARS-CoV-2 variants of concern, including several Omicron sublineages. *Med. Microbiol. Immunol.* **212**, 307–322. 10.1007/s00430-023-00774-9 (2023). 37561226 10.1007/s00430-023-00774-9 PMC10501957

[25] Deng, X. et al. Genomic surveillance reveals multiple introductions of SARS-CoV-2 into Northern California. *Science* **369**, 582–587. 10.1126/science.abb9263 (2020). 32513865 10.1126/science.abb9263 PMC7286545

[26] Grubaugh, N. D. et al. Genomic epidemiology reveals multiple introductions of Zika virus into the United States. *Nature* **546**, 401–405. 10.1038/nature22400 (2017). 28538723 10.1038/nature22400 PMC5536180

[27] O’Toole, A. et al. Assignment of epidemiological lineages in an emerging pandemic using the pangolin tool. *Virus Evol.* **7**, veab064. 10.1093/ve/veab064 (2021). 34527285 10.1093/ve/veab064 PMC8344591

[28] Aksamentov, K., Roemer, C., Hodcroft, E. B. & Neher, R. A. Nextclade: clade assignment, mutation calling and quality control for viral genomes. *J. Open Source Softw.* **6**, 3773 (2021).

[29] Wadford, D. A. et al. Implementation of California COVIDNet—A multi-sector collaboration for statewide SARS-CoV-2 genomic surveillance. *Front. Public Health* **11**, 1249614. 10.3389/fpubh.2023.1249614 (2023). 37937074 10.3389/fpubh.2023.1249614 PMC10627185

[30] R Core Team. R: A language and environment for statistical computing, (R Foundation for Statistical Computing, 2021), https://www.R-project.org/.

[31] Wickham, H. *Ggplot2: Elegant Graphics for Data Analysis* (Springer, 2009).

[32] Tegally, H. et al. Emergence of SARS-CoV-2 Omicron lineages BA.4 and BA.5 in South Africa. *Nat. Med.* **28**, 1785–1790. 10.1038/s41591-022-01911-2 (2022). 35760080 10.1038/s41591-022-01911-2 PMC9499863

[33] Ma, K. C. et al. Genomic surveillance for SARS-CoV-2 variants: Circulation of Omicron lineages—United States, January 2022–May 2023. *MMWR Morb. Mortal Wkly. Rep.* **72**, 651–656. 10.15585/mmwr.mm7224a2 (2023). 37319011 10.15585/mmwr.mm7224a2 PMC10328465

